# The complexity-theoretic Bell inequality

**DOI:** 10.1093/nsr/nwy075

**Published:** 2018-07-24

**Authors:** Zhengfeng Ji

**Affiliations:** Centre for Quantum Software and Information, Faculty of Engineering and Information Technology, University of Technology Sydney, Australia

‘I am a quantum engineer, but on Sundays I have principles.' John Stewart Bell once started a colloquium with these words. The Bell inequality, one of the most important and fundamental results of quantum physics, probably originates from a flash of insight he had on a Sunday afternoon around 1964. The inequality proves Einstein wrong and provides a concrete method to tell apart quantum mechanics and classical theories. A variant of the Bell inequality, known as the CHSH inequality named after John Clauser, Michael Horne, Abner Shimony, and Richard Holt, states that |*S*| ≤ 2 in any classical theories where

\begin{equation*}
S = \langle A_0 B_0 + A_0 B_1 + A_1 B_0 - A_1 B_1 \rangle ,
\end{equation*}

and *A*_*i*_, *B*_*j*_ are observables; however, quantum mechanically it is possible to violate the inequality and have $\left|S \right| = 2 \sqrt{2}$.

Forty years later, a group of computer scientists, Richard Cleve, Peter Høyer, Ben Toner and John Watrous [1], provided a computer science perspective on Bell inequalities in the framework of multi-player one-round games. In a multi-player one-round game, a referee samples and asks questions to multiple non-communicating players and checks the validity of the answers according to some rule. The classical value of the game is the maximum success probability with which the players can pass the referee's check. Multi-player one-round games form a well-studied topic in theoretical computer science and have connections to constraint satisfaction problems, interactive proof systems, probabilistically checkable proof (PCP) and the celebrated PCP theorem. Cleve *et al.* reformulated Bell inequalities as multi-player one-round games such that the players can achieve a higher success probability, called the quantum value, when they measure a shared entangled state to obtain the answers. They also pointed out that shared entanglement may cause the so-called soundness problem in quantum interactive proof systems, an intriguing phenomenon that leads to a series of investigations on the limitation and power of entanglement in multi-player games.

It is well known that it is NP-hard to approximate the classical value of a game to inverse polynomial precision where NP stands for nondeterministic polynomial time. Just how hard is it to approximate the quantum value of a game? We do not know the answer after a decade of research efforts, mainly because the players measure on an arbitrarily high dimensional quantum system. Because of the soundness problem pointed out by Cleve *et al.*, we did not even know how to establish that the quantum value is NP-hard until Kempe *et al.* finally proved it in 2007 [2]. Very recently, the author established that the quantum value of a game is NEXP-hard to approximate [3,4] where NEXP stands for nondeterministic exponential time. This can be seen as a *complexity-theoretic Bell inequality*. The standard Bell inequality tells us that the classical and quantum values can be different for a fixed game; our result instead establishes that the computational complexities for approximating the classical and quantum values are different, since NP ≠ NEXP.

The complexity of Bell inequalities or the quantum values of multi-player one-round games has deep connections to quantum physics and theoretical computer science as we have already discussed. It is also related to the Connes embedding conjecture in mathematics. Using a group theory approach, Slofstra proved that the problem of determining whether a game has quantum value 1 or not is undecidable [5]. Is the quantum value approximation problem also undecidable or can we establish any complexity upper bound? Only time will tell.
